# Supplementary material for: Are carbon emissions trading and green financial instruments synergistic? -Comprehensive quantitative research based on content analysis
Source: PLoS One. 2024 Mar 7;19(3):e0298601. doi: 10.1371/journal.pone.0298601 (PMC10919601; doi:10.1371/journal.pone.0298601)
Supplement: S1 Data — (ZIP) [file pone.0298601.s001.zip › Data/地级市Z_F工作报告.zip!/░▓╗╒/║╧╖╩╩╨/└┤╫╘╣1⁄2╓┌║┼í╛┬φ┐╦╩2╛▌═°í┐_2012.docx]

# **2012年政府工作报告**

发布时间：2012-01-10 10:10

【字体：[大](http://www.hefei.gov.cn/public/1741/javascript:void(0);) [中](http://www.hefei.gov.cn/public/1741/javascript:void(0);) [小](http://www.hefei.gov.cn/public/1741/javascript:void(0);)】

****政府工作报告****

各位代表：

现在，我代表市人民政府，向大会作工作报告，请予审议，并请政协委员和其他列席人员提出意见。

一、过去五年及2012年工作回顾

本届政府任期的五年，我们以科学发展观为指引，在省委省政府和市委的坚强领导下，奋力抢抓中部崛起、产业转移等重大机遇，积极应对复杂多变的宏观经济环境，紧紧围绕大发展大建设大环境的工作主题，深入实施工业立市、县域突破、创新推动、东向发展、可持续发展战略，完成了各项既定目标任务，实现了经济社会又好又快发展，取得了跨越赶超、争先进位的辉煌业绩，把合肥发展推上了一个更高的起点。

——这五年，是综合实力大幅跃升的五年。全市生产总值达到4100亿元以上，由全国26个省会城市第18位升至第15位，年均增长16%以上，成为全国发展最快的省会城市。规模以上工业总产值达到6600亿元；增加值1650亿元、年均增长23.7%，总量由省会城市第15位升至第11位。财政收入达到694.4亿元、年均增长24.6%，其中，地方财政收入达到389.5亿元、年均增长28.3%，总量进入省会城市前10位。全社会固定资产投资突破4000亿元，总量由省会城市第9位升至第7位，累计完成15300亿元、年均增长29.6%。社会消费品零售总额达到1290亿元、年均增长20%，总量由省会城市第18位升至第16位。城镇居民人均可支配收入达到25400元，农民人均纯收入达到9200元，年均分别增长13.6%和17.2%。

——这五年，是产业层次显著提升的五年。坚持走新型工业化道路，建立完善“四大政策体系”，加快发展与转型发展同步并进。推进信息化与工业化融合发展，成为国家级两化融合试验区和电子信息国家高技术产业基地。工业投资累计完成5438亿元、年均增长37.2%，其中技改投资2872亿元。京东方6代线、大陆轮胎、联想产业基地等一大批标志性大项目落地生效，家电、汽车、装备制造、食品及农产品加工等支柱产业主导作用更加突出，新型平板显示、太阳能光伏、新能源汽车、公共安全等战略性新兴产业占据全国领先地位。建筑业发展壮大，增加值占全市生产总值12%以上。服务业蓬勃发展，增加值达到1600亿元、年均增长12%。金融体系日趋完善，本外币各项贷款余额突破6000亿元，上市公司达到30家、居省会城市第9位，滨湖国际金融后台服务基地入驻机构达到14家；现代商贸业提质扩量，入选国家现代物流技术应用和共同配送试点城市，电子商务、服务外包、连锁超市等快速发展；会展经济长足发展，跻身中国“十大节庆城市”；旅游业增长迅速，年收入突破400亿元。文化产业实现增加值260亿元，占全市生产总值比重超过6%。

——这五年，是城市建设快速拓展的五年。行政区划调整圆满成功。坚持规划引领、交通先行，老城提升、新区开发、组团展开同步推进，大建设如火如荼，累计完成1629项工程、总投资1886亿元，建成道路1035公里、桥梁134座，城市建成区面积扩大到360平方公里，城区人口突破350万，城镇化率达到65%。区域性综合交通枢纽加速建设，新桥国际机场全面建成，合蚌高铁开通运营，高速公路形成“一环六射”格局，航运通江达海能力得到提升。轨道交通一号线全面建设、二号线正式开工。市区道路形成“两环七射加方格网”，主城区与各组团实现“一刻钟快速交通”。滨湖新区路网围合面积34平方公里，常住人口突破40万；政务文化新区全面建成；各组团产城一体加快推进。市政公用事业全面发展，水电气热等供应能力显著提升，公交车数量增至近4000标台。环境保护和生态建设成效明显，新建、续建11座污水处理厂，建成城区污水主干管1628公里、覆盖率达98%，基本实现污水全收集全处理，巢湖总体水质由劣V类转为V类，城市建成区绿化覆盖率增至45.2%，全市森林覆盖率达到22.2%。

——这五年，是农村面貌发生巨变的五年。坚持“三化同步”，城乡统筹、融合发展迈出坚实步伐，新农村建设走在全省前列。县域经济发展迈上快车道，总量达到1300亿元、年均增长16.2%，肥西跻身全国百强，肥东、长丰进入中部百强，巢湖、庐江大建设“十大工程”初显成效、发展提速提质。农业转型升级成效明显，土地流转突破200万亩，蔬菜瓜果面积达到180万亩，农业科技进步贡献率达到58%，肥西苗木花卉、长丰草莓等特色产业享誉全国。农业产业化经营水平跃上新台阶，农民专业合作组织超过2000家，年销售收入10亿元以上龙头企业达到14家，新增庐江郭河等省级以上现代农业园区40个。进一步加大对“三农”的投入，农村基础设施全面改善，公路通车里程达到1.46万公里，村村通水泥（油）路；农村防洪保安、水源保障能力显著增强，建成一批重点水利工程；“清洁家园、绿化乡村”富有成效，整治村庄497个，改造危房161.3万平方米，建设集中居住和农民新型社区440万平方米；土地综合整治整村推进成为全国样板。

——这五年，是科技创新实现突破的五年。大力推进国家创新型试点城市、合芜蚌自主创新综合试验区建设，示范区“一中心、三基地”一期工程全面建成。城市基础科研实力跃居全国前列，创新发展形成领先优势。改进科学技术进步奖励办法，设立市政府质量奖。深入实施创新型企业培育计划，高新技术企业增至615家，居全国省会城市第8位；工程（技术）研究中心、企业技术中心、重点实验室等研发机构增至680家。建立公共安全、新能源汽车等十大战略性新兴产业研究院。全社会科技研发投入占全市生产总值2.3%，高新技术产业增加值占全市生产总值22%，在全国同类城市中均居前列。科技金融试点取得新进展，引进一批国内外知名风投创投机构。大力实施合芜蚌人才特区等人才工程，建成一批留学生创业园、“海智”基地和院士工作站。加强自主品牌建设，成为首批国家商标战略实施示范城市。

——这五年，是改革开放纵深推进的五年。相继实施“十九项改革”、“八项开放”举措，充分释放发展活力。新一轮国企开放式改革重组取得突破，非公有制经济加快发展，各类市场主体超过30万户。“六分开”的城市建设管理体制更加完善，“借、用、还”一体的投融资新体制良性运转，招投标管理体制成为走向全国的“合肥模式”，节约集约用地试点市建设成效显著。推进全省城乡一体化综合配套改革试验区建设，农村改革取得新进展。大力推进行政审批制度改革，率先开门“三定”政府机构职能、编制预算和实行行政处罚群众公议、听政等制度，效能革命和政务公开全面深化，公务员转任实现法制化、常态化。加强承接产业转移示范区建设，坚持大招商、招大商，累计招商引资6880亿元、年均增长29.7%，新引进境外世界500强企业13家，与央企、知名民企合作发展取得重大成效。成功承办中博会、自主创新要素对接会、家博会等重大经贸展会，大力支持“合肥之友”打造国际化招商平台。对外贸易不断扩大，累计进出口额达到529亿美元、年均增长16.4%。成为长三角城市经济协调会成员和长江沿岸中心城市经济协调会常任主席方。加快合肥经济圈一体化建设，积极推进与六安市霍邱县、寿县结对合作，阜阳合肥现代产业园区加快建设。援疆、援藏工作取得新成效。

——这五年，是群众生活明显改善的五年。坚持从发展和创富的角度抓民生，不断增进人民福祉。累计民生工程投入211.7亿元，完成项目32269个，惠及700万人次以上。新建、改扩建各类中小学校、幼儿园近千所，校舍安全工程全面完成，义务教育均衡发展、推进素质教育和学前教育改革试点走在全国前列；职教城初具规模，地方高等教育迈上新台阶。成功创建国家级创业型城市，新增城镇就业56.8万人，动态消除“零就业家庭”。社会保险体系日益健全，各类保险待遇稳步提高。城乡救助体系基本形成，实现城乡低保、医疗保险和合作医疗、困难群众医疗救助全覆盖。建立价格调节基金制度，完善价格上涨与特困群体生活补贴联动机制，价格总水平保持基本稳定。整治老旧小区157个，改造城中村、危旧房75个，建设各类保障性住房15.8万套，城镇居民人均住房面积增至36.7平方米。实施农村饮水安全工程328处，解决153.7万人饮水安全问题。基层医药卫生体制改革创全国样板，医疗卫生资源大幅扩充。四级公共文化服务网络基本形成，渡江战役纪念馆等一批大型公益文化设施建成开放，歌曲《追寻》获全国“五个一工程奖”，成功承办第20届全国金鸡百花电影节等重大文化活动。第四届全国体育大会、第十五届亚洲轮滑锦标赛等全国性、洲际赛事圆满成功，参赛奥运会、亚运会等取得佳绩，全民健身运动蔚然成风。

——这五年，是社会管理不断创新的五年。扎实推进法治合肥、平安合肥、和谐合肥建设，保持政通人和、稳定有序的良好局面。坚持依法行政，自觉接受人大法律监督和政协民主监督，累计办结人大代表议案和建议、政协提案3019件。建立项目推进机制，顺利完成全国社会管理创新综合试点任务。完善治安防控体系，八类案件发案率位列省会城市最低水平，五获全国社会治安综合治理优秀城市，再次确认“长安杯”。积极推行重大决策项目社会稳定风险评估，深入开展“大接访”，完善矛盾纠纷排查化解机制。全面推进网格化、组织化、信息化、服务化，和谐社区建设成效明显。应急管理机制逐步建立，应对各类突发事件能力进一步增强。深入开展重点行业和“三车”交通安全等专项整治，强力推进乡镇安全监管规范化、企业安全标准化建设，安全生产形势持续稳定。文明创建深入开展，四次蝉联创建全国文明城市工作先进市。加强国防动员和后备力量建设，实现全国双拥模范城“七连冠”。人防工程及民防建设走在全国前列。人口计生、防震减灾、民族宗教、外事侨务、地方志、科普、气象、统计、档案、保密、社科等工作水平实现新提升，工会、共青团、妇女儿童、老年人、残疾人和关心下一代等工作取得新进步。

刚刚过去的2012年，面对经济下行的压力，我们牢牢把握稳中求进的工作总基调，全面贯彻落实党的十八大和省市党代会精神，主动调整转型，保持发展势头，实现争先进位。预计，全市生产总值增长13.5%以上；规模以上工业增加值增长17%以上；财政收入增长11.3%，其中，地方财政收入增长15%；全社会固定资产投资增长23.6%；社会消费品零售总额增长16.5%以上；城镇居民人均可支配收入增长13%以上，农民人均纯收入增长17%；城镇登记失业率控制在4.5%以内；居民消费品价格指数上涨2.4%。

一是加强经济运行调节。出台38条促进经济平稳较快发展的政策措施，加大对企业帮扶支持的力度。新开工亿元以上项目420个，完成投资800亿元。江汽-纳威司达发动机、泥河铁矿等项目获得核准；鑫晟8.5代线、合肥电厂#6机组等开工建设；晶弘电器、晶澳太阳能一期等建成投产。与国开行等金融机构战略合作取得新成效，新增贷款超过1000亿元。美亚光电挂牌上市，另有3家待发、9家在审；鑫城、海恒、高新集团、巢湖城镇投、肥西桃花工业园、合肥中小企业等6支企业债券获批发行。积极争取各类建设用地指标，确保重大项目需求。存量建设用地升级改造三大政策效果明显，房地产市场保持稳定。大力帮助骨干企业解决生产经营难题，积极引导光伏企业开拓西部市场。

二是加快产业转型发展。实施工业“新跨越、进十强”、中心城区工业优化布局转型发展、百家高成长性企业培育工程等政策意见，开展质量强市活动和标准化等工作，推动支柱产业转型升级、新兴产业加速壮大。六大支柱产业完成产值3850亿元、占工业总产值58.3%，战略性新兴产业实现增加值425亿元、增长21%。现代服务业集聚发展，华南城等一批重大项目成功签约。开工建设中科大先进技术研究院，谋划筹建合工大智能制造技术研究院，首批国家级文化和科技融合示范基地挂牌，全球首个46节点的城域量子通信网运行，“十城千辆”、“十城万盏”、“金太阳”等示范应用推广工程走在全国前列。

三是提升城市建设和管理水平。编制了城市空间发展战略及环巢湖地区生态保护修复与旅游发展、半汤和汤池国际温泉旅游度假区等重大规划。新一轮市、县、乡三级土地利用总体规划分别获国务院和省政府批准。全年新建、续建大建设工程840项，完成投资217亿元。铁路枢纽南环线、高铁南站以及合马路、军二路等改造工程加快推进；机场高速、南淝河大桥等建成通车；阜阳北路、铜陵路等高架桥建设进展顺利。铺设污水管网210公里，完善排水设施网格890个；六水厂二期、七水厂一期、燃气应急调峰工程等快速推进；固定电话号码完成升8位；提高了公交IC卡折扣率；新建老城区停车位7000个。掀起“三城同创”热潮，全国文明城市创建测评取得优秀成绩。查处违法建设和整治“五小行业”、砍树毁绿、有烟烧烤、户外广告、“黑头车”等取得实效。

四是启动美好乡村建设。出台美好乡村建设实施意见，深入推进“三新工程”和“亮化工程”。启动实施董铺和大房郢水库水源地、庐江汤池、新桥国际机场周边三大土地整治项目，规范展开城乡建设用地增减挂钩试点和工矿废弃地复垦试点。粮食生产实现“九连增”，环城都市、环湖观光、丘陵生态等三大农业产业带建设初见成效，新建万亩现代农业示范区31个、各类现代农业科技示范园区18个，完成植树造林24.4万亩。疏堵并举推进秸秆禁烧，秸秆综合利用率达到80%。开展“农田水利建设高潮年”活动，重点实施了水库加固、圩堤达标、灌区配套等十大工程。

五是推进巢湖治理开发。《巢湖流域水污染防治“十二五”规划》和国家水生态系统保护与修复试点获批实施。扎实推进巢湖综合治理“八大工程”；高起点规划建设环巢湖生态示范区，首批113个项目开工建设，总投资超过500亿元。环巢湖道路全面动工、北岸建成通车；巢湖闸复线工程、裕溪船闸扩建工程建成试航；兆西河整治及黄湾闸工程开工；234个环巢湖生态农业项目启动实施。

六是加大改革开放力度。国通管业等一批国有企业完成战略重组，建投集团、巢湖城投、科农行等完成增资扩股，国有资产实现保值增值。出台企业股权和分红激励试点“6+1”政策，32家企业参加试点。加快实施滨湖新区、合巢经开区管理体制改革，顺利完成市政和园林管理体制下划。市招投标中心年交易额突破1000亿元。稳步推进“营改增”试点。全面推开县级公立医院改革，药品全部实现零差率销售。农村商业银行基本实现全覆盖，农村产权抵质押融资试点进展顺利。“百名县处级领导干部大招商”活动深入开展，赴境内外系列招商推介取得实效，全年完成招商引资2102亿元，引进工业和现代服务业大项目87个。进出口总额达到165亿美元、增长34%。合肥出口加工区正式封关运行，申报设立综合保税区取得重大进展。

七是扎实推进社会建设。“33+7”项民生工程全面推进，各级财政投入76亿元。启动公建幼儿园“管办分离”改革试点，在全国率先取消城区普通高中择校，在全省率先免除义务教育阶段学生书本费。新增城镇就业14万人。社会保障提标扩面，城乡居民养老保险制度实现全覆盖；城乡居民医保和新农合统筹并轨，报销比例提高到55%以上。建设各类保障性住房7.53万套，实施城中村、危旧房改造47个，整治老旧小区36个。改革物业综合服务收费管理，80%的居民小区实行“一费制”。首批4个省市重大文化项目启动建设；第七届中国曲艺牡丹奖比赛、市十运会等重大文体活动圆满成功；《合肥通史》启动编撰，《合肥市志》即将发行。大力开展重点隐患排查整治，加快烟花爆竹生产企业有序退出。重拳打击非法传销成效明显，食品药品等市场秩序进一步规范。

八是积极改进政务服务。加强政府立法，提请市人大常委会审议地方性法规2件，制定、修改政府规章5件。完善《政府工作规则》，出台《政府重大行政决策程序规定》。在全国省会城市率先推进政务服务中心标准化建设、整合开通“12345政府服务直通车”。建立重大项目驻点监察制度，推行建设工程竣工联合验收。创新开展“问政合肥-政风行风面对面”媒体联动活动，积极引导网络舆情。实施行政执法自由裁量权试点，全面推开廉政风险防控管理工作，建立县处级干部廉政档案，健全经济责任审计管理制度，加大行政问责力度。加强增收节支，“三公”经费保持“零增长”。强化政策谋划和储备，严格精简会议、文件、简报和领导同志事务性活动。

各位代表！

五年来，我们始终以忠于人民的高度自觉和敢为人先的创新精神，走出了一条符合市情的科学发展之路，既创造了丰硕的物质成果，也积累了宝贵的精神财富，从合芜蚌自主创新综合试验区到皖江城市带承接产业转移示范区，从国家创新型试点城市到国家企业股权和分红激励试点，从国家节约集约用地试点市到全国社会管理创新试点市，推出了一批在全国具有示范作用的“先行之作”，先后获得全国科技进步先进市、全国创业先进城市、全国文化体制改革先进地区、全国推进义务教育均衡发展先进地区等75项国家级或全国性荣誉，极大地增强了全市人民的自豪感和归属感，显著地扩大了合肥在全国的知名度和影响力，不断地提升了合肥在全国发展格局中的地位和作用。

各位代表！

回顾五年成绩，我们倍感自豪！这些成绩的取得，是党中央国务院、省委省政府和市委坚强领导的结果，是市人大、市政协及社会各界大力支持的结果，是全市广大干部群众顽强拼搏的结果。在此，我谨代表市人民政府，向全市广大工人、农民、知识分子、干部、驻肥解放军指战员、武警官兵和政法干警，向各民主党派、工商联、人民团体和社会各界人士，向所有关心支持合肥改革开放与现代化建设的海内外朋友，表示衷心的感谢，并致以崇高的敬意！

同时，我们也要清醒看到，发展中还存在许多不足和问题，主要是：综合实力仍然不够强，产业层次不够高，服务业发展相对滞后；资源环境约束趋紧，节能减排压力加大，巢湖综合治理开发任务艰巨；城乡居民收入水平还有待进一步提高，基本公共服务不够均衡，社会矛盾不断增多，社会管理难度加大；民营经济和中小企业发展不够充分，开放型经济发展水平不高，国际化程度较低；城区经济转型不快，县域经济实力不强，城乡统筹、区域协调发展任务繁重；政府职能转变、效能提升还存在薄弱环节，一些领域消极腐败现象还不同程度存在。对这些不足和问题，我们要高度重视，认真加以解决。

二、今后五年的目标任务

综合判断，复杂多变的宏观经济环境将长期存在，但合肥发展处于大有可为的黄金发展期没有变，工业化、城镇化加速推进的阶段性特征没有变，加速崛起的大趋势没有变。今后五年，我们要在新一轮世界工业革命的起跑线上抢抓先机、占据前沿，在全国区域发展格局战略调整中勇挑重担、提升地位，在安徽“三个强省”建设中示范引领、多作贡献，全面建成小康社会，开创建设现代化新兴中心城市的新局面。这是时代赋予我们的历史使命，这是省委省政府授予我们的政治责任，这是全市人民寄予我们的热切期待。只要我们全面把握机遇，沉着应对挑战，就一定能够争取主动、增创优势、赢得未来，就一定能够实现建设美好合肥的宏伟蓝图！

今后五年，政府工作的指导思想是：高举中国特色社会主义伟大旗帜，以科学发展观为指导，深入贯彻落实党的十八大和省市党代会精神，紧紧围绕“新跨越、进十强”的总体要求和“大湖名城、创新高地”的战略定位，深化改革开放，强化创新驱动，转变发展方式，建设生态文明，加强民生保障，统筹推进工业化、信息化、城镇化和农业现代化，加快建设现代化新兴中心城市，并朝着在全国有较大影响力的区域性特大城市方向阔步迈进，在美好安徽建设中充分发挥核心辐射带动作用，率先全面建成小康社会。

今后五年的主要奋斗目标是：全市生产总值达到8500亿元、年均增长12%以上；财政收入年均增长10％；规模以上工业增加值年均增长14%以上；全社会固定资产投资年均增长16%；社会消费品零售总额年均增长15%；居民收入增幅高于经济增幅；人口年均自然增长率控制在7‰以内；节能减排目标任务全面完成。

今后五年，我们要着力在七个方面实现新跨越：

在现代产业基地建设上实现新跨越。坚持“新优融合、高端引领、做大总量、加速转型”，着力打造6个千亿元、若干个五百亿元级产业，培育发展3~5家产值超500亿元、50家产值超百亿元的龙头企业集团，发展壮大一批“精、优、特、新”的中小企业。加快发展电子信息及新型平板显示、新能源及太阳能光伏、新材料、高端装备制造、新能源汽车、节能环保、生物、公共安全等战略性新兴产业，提升发展家电、汽车、装备制造、食品及农产品加工等优势主导产业，全面建成全国重要的现代产业基地。推动生产性服务业集聚化、生活性服务业便利化发展，建成一批特色现代服务业聚集区，形成区域性金融、物流、会展、商贸、旅游、信息服务中心。大力发展现代设施农业、优质高效农业、绿色生态农业、休闲观光农业，建设一批现代农业产业园，加快农业现代化进程。

在城市建设管理上实现新跨越。坚持走集约、智能、绿色、低碳的新型城镇化道路，以“1331”的市域空间布局为引领，进一步完善城镇体系，城镇化率达到75%。优化提升主城区，积极发展副中心城市和新产业基地，分类建设特色小城镇。中心城区面积拓展到500平方公里，常住人口达到500万。滨湖新区建成全省行政中心、文化中心和金融商务中心。全面完成二环以内老旧小区整治和城中村、旧城改造。完善新桥国际机场功能，建成合福、商合杭客运专线和庐铜铁路，加快推进合六、合巢芜、合安、合宁等城际铁路建设，升级改造合铜、合安、合淮、合六等国省干线公路，完成“五河一湖”航道网建设，轨道交通建成一、二号线、启动中长期建设，城市路网进一步完善，形成全国重要的综合交通枢纽。争创全国文明城市，加快建设智慧城市、信用城市，推进城市管理科学化、法制化、规范化，打造整洁、有序、美观、舒适的城市人居环境。

在自主创新上实现新跨越。加快国家创新型试点城市、合芜蚌自主创新综合试验区建设，着力构建以企业为主体、市场为导向、产学研用相结合的技术创新体系。加强协同创新，强化人才支撑，大力推进与名校、大所的战略合作，着力打造“中科智城”，形成中国科技创新资源集聚发展的新高地。加大名牌产品、驰（著）名商标的培育和保护，打造一批具有自主知识产权和核心竞争力的合肥品牌。高新技术产业产值突破9000亿元，累计发明专利授权量突破万件，全社会科技研发投入占全市生产总值3.3%，在全国率先建成创新型城市。

在改革开放上实现新跨越。深化国有企业改革，加快产权多元化和资源整合步伐。制定特殊政策，采取有效措施，全面优化环境，推动非公有制经济大发展。大力推进投融资、财税、金融、土地、招投标等改革，建立健全统一开放的资本、技术和人力资源等各类要素市场。深化户籍、就业、农村产权等制度改革，加快农民市民化进程。深化行政体制改革，加快推进教育、文化、卫生等事业单位分类改革。抢抓中部崛起的战略机遇，加强承接产业转移示范区建设，进一步扩大招商引资，加强与央企、知名民企、跨国公司和上市公司的合作发展，拓展和深化区域合作发展的领域和内容，支持有条件的企业“走出去”，大力提升国际化发展水平。加快推进合肥经济圈一体化发展，打造具有全国影响力的都市圈品牌。

在城乡一体发展上实现新跨越。完善强农惠农富农政策体系，在发展规划、产业布局、基础设施、基本公共服务等方面加大统筹力度，形成以工促农、以城带乡、工农互惠的长效机制，打造全省乃至全国城乡一体化发展示范区。加强县城和重点镇规划建设管理，提升产业发展、公共服务、吸纳就业、人口集聚功能。全面推开美好乡村建设，大规模开展土地整治，加快建设一批布局合理、环境优美、设施配套、功能完善的新型农村社区。大力推进新一轮扶贫开发，加快贫困乡村发展。全面推动县域经济发展取得新突破，肥西力争进入全国百强县50强，肥东、长丰、巢湖、庐江力争进入全国百强。

在生态文明建设上实现新跨越。坚持生态环保优先和资源节约集约利用，着力推进绿色发展、循环发展、低碳发展，建设全国著名的生态城，打造天蓝、地绿、山青、水净的美好家园。环巢湖生态示范区建设争取上升到国家战略层面，着力打造城湖共生、生态宜居的典范。坚持“治湖先治河、治河先治污”，有效控制和消减点源面源内源污染，实现巢湖水环境持续改善。依托湖光山色、历史文化等资源，加快建设以大湖、温泉、湿地、名镇等为特色的国际滨湖度假旅游目的地。大力创建国家生态园林城市、国家森林城市，扎实推进“森林增长工程”和城区绿化美化工程，城市绿化覆盖率达到46.5%，全市森林覆盖率达到30%以上。

在幸福合肥建设上实现新跨越。坚持以人为本、富民优先，大力推进以保障和改善民生为重点的社会建设，不断提高全市人民的幸福感。大力实施城乡居民收入倍增计划，努力增加居民工资性、经营性、财产性、转移性收入。加大民生投入，加快构建终身教育、就业服务、社会保障、医疗卫生、住房保障、社会养老等服务体系，促进基本公共服务均等化、优质化。以重大项目、重大活动、重大展会为抓手，完善公共文化服务体系，大力发展文化产业，推动文化与科技、旅游、体育等深度融合，加快建设文化强市。全面推进社会管理创新，着力构建公共安全体系，全力维护社会和谐稳定，努力建成全国社会管理示范城市。

三、2013年政府工作

2013年，是全面贯彻党的十八大精神的第一年，是新一届政府的开局之年，是实施“十二五”规划承前启后的关键之年，做好今年工作意义重大。我们要坚持以科学发展观为指导，以提高经济增长质量和效益为中心，按照“平中见奇、稳中求快、调整转型、夯实基础”的要求，保持经济持续健康较快发展和社会和谐稳定，扎扎实实开好局、起好步。

综合考虑，2013年经济社会发展主要目标是：全市生产总值增长12%以上；财政收入增长10%，其中地方财政收入增长8%；全社会固定资产投资增长20%以上；规模以上工业增加值增长14%以上；社会消费品零售总额增长15%；城镇居民人均可支配收入增长13%，农民人均纯收入增长14%；城镇登记失业率控制在4.5%以内；人口自然增长率控制在7‰以内；居民消费价格指数涨幅控制在3.5%左右；群众安全感指数保持在90%以上；节能减排达到省控目标。

今年，要着力做好十个方面工作：

（一）扩大有效需求

加大投资力度。围绕巢湖综合治理开发、支柱产业、新兴产业、城乡基础设施、节能环保、民生工程等重点领域，谋划和实施一批超50亿元的重特大项目，不断优化投资结构。加强省“861”、市“1346”行动计划重大项目调度，确保完成全社会固定资产投资4600亿元。推进银企对接，深化与国开行等战略合作，引导各类金融机构扩大信贷投放，全年新增间接融资1200亿元。鼓励支持企业发行债券、短期融资券、中期票据等，大力实施“百家企业上市工程”，力争直接融资400亿元以上。通过市县联动、推动金融创新，打通县（市）融资途径。加强与深圳前海合作，开辟境外人民币回流的有效渠道。深化节约集约用地试点，用足用好年度用地指标，大力实施“增减挂”和工矿废弃地复垦等，深入推进存量建设用地挖潜。

努力开拓市场。认真落实品牌促进、节能补贴等扩大消费政策，促进汽车、家电、太阳能光伏等地产品销售。着力推进连锁经营、集中配送，提升“万村千乡”和“新网”工程，培育文化体育、旅游休闲等新兴消费。广泛开展农超、农校等对接，加快粮食产业化载体建设，完善便民惠民蔬菜流通体系。制定开放型经济发展政策，设立支持企业“走出去”专项资金，加快出口加工区建设，争取获批设立综合保税区，全年实现进出口总额180亿美元。

优化企业服务。完善“四大政策体系”，落实各项扶持政策，加强政策谋划储备，进一步增强政策的超前性、针对性和有效性。实施结构性减税，清理各项收费项目，切实减轻企业负担。加强煤电油气运等调度，保障企业正常生产经营。深入企业开展帮扶，一企一策缓解骨干企业经营困难，多措并举解决中小微企业融资、用地、用工等难题。

（二）做大做强产业

做强优势产业。大力推进家电、汽车、装备制造、食品及农副产品加工等优势主导产业加速向产业链两端延伸、价值链高端攀升，增强核心竞争力。全面建成国家家用电器产品质检中心。加快TCL冰洗生产基地、江汽-纳威司达发动机、广银铝业、南车轨道车辆制造维修等一批重大项目建设，力争再打造1~2个千亿级产业集群。

壮大新兴产业。实行分类指导，量身定制扶持政策，一手抓企业发展和项目建设，一手抓示范和应用推广，加快鑫晟8.5代线、豪威光电、彩虹蓝光LED等重特大项目建设，推进量子通信、新型平板显示、半导体、云计算、集成电路等电子信息全产业链发展，加快太阳能光伏、新能源汽车、智能装备等产业发展壮大，全年战略性新兴产业产值达到2000亿元、增长20%以上。积极申报国家新型显示、硅基功能材料、智能装备制造等新兴产业集聚发展试点。

提升服务业。加快滨湖国际金融后台服务基地建设，积极引进各类金融保险、证券基金、风投创投等来肥设立分支机构。优化商圈规划布局，建设一批特色街区和功能区，打造一批高端商贸综合体。围绕建设全国流通领域现代物流示范城市，加快华南城、南翔万商、宝湾物流、农产品国际物流园等重大项目建设。加快发展环巢湖旅游，大力推进万达文化旅游城、国际帆船俱乐部等重大项目建设。围绕创建国家电子商务示范城，着力打造一批产业集聚区。大力发展会展经济，全年举办各类大型展会170场以上。围绕建设服务外包示范城市，重点打造呼叫、共享等服务中心。

推进园区转型发展。制定转型发展的政策措施，促进园区优化升级。按照产城一体的要求，大力推动经开区、高新区、新站区、合巢经开区等四大开发区完善功能、提升品质、持续发展。加快都市产业园建设，每个城区兴建一批标准化厂房，大力发展移动互联网、物联网、总部经济以及研发设计等新业态，形成都市产业发展新高地。加快肥东经开区、肥西桃花工业园、长丰双凤工业园扩容升级步伐，加强居巢经开区、庐江经开区等基础设施建设，进一步提升县域工业园区产业承载力和产业集中度。

（三）推进城市建设

加快综合交通枢纽建设。启用新桥国际机场。制定新一轮城市轨道交通建设规划，加快一号线建设，全面推进二号线建设，积极启动中长期建设前期工作。积极推进商合杭客运专线、庐铜铁路和合宁、合安、合六等城际铁路前期工作。加快铁路枢纽南环线、高铁南站及枢纽配套工程、合福客运专线以及店埠河、兆河、合裕航道升级改造、合肥港综合码头二期等工程建设。开工建设合铜、合淮、合安路等升级改造工程，推进合六路、军二路、环巢湖道路等建设，力争完成合马路、206国道改线段等主体工程，加快中心城区与副中心城市快速交通建设。

加强市政设施建设。完善城市骨干路网，建成铜陵路高架、阜阳路高架、怀宁北路等工程，打通望江西路，启动繁华大道东延、铜陵北路、肥西路等主次干道建设。加强微循环交通改造，推进一批支路工程建设，改造一批城区小街巷。加强城市慢行设施规划建设，加快建设老城区停车场，启动地下空间开发。大力创建“国家公交都市”，新增公交运力600台，适量投放一批城市出租车。积极推进龙河口引水工程，建成六水厂二期和七水厂一期工程。大力推进“气化合肥”，加快车辆“油改气”推广示范、分布式能源和管道天然气项目建设。

提升城市管理水平。全面建成“数字城管”工程，全力推进城区城市管理重心下移，进一步发挥街道主体作用，强化网格管理，夯实城市管理基础。按照“一街一主题、一路一特色”的要求，开展试点路段立面景观整治，推出一批示范街、特色街。继续保持打击非法营运的高压态势，加大火车站等重点区域综合整治力度。提升摊点精细化管理水平，打造一批特色高品质夜市、摊点群。巩固完善新型物业管理机制，积极改进无物管小区管理办法。

（四）加强自主创新

提升企业创新能力。完善企业初创、成长、发展等不同阶段的支持政策，分类实施创新领航企业、创新小巨人、科技型小微企业培育计划，全年新增高新技术企业、创新型企业100家，培育“创新小巨人”企业100家，新建企业工程（技术）研究中心、重点实验室、检测中心等研发机构100家，孵化初创型科技企业100家，实施重点产学研合作项目100项，转化科技成果100项。

大力推动协同创新。建成中科大先进技术研究院一期工程，组建一批国家联合实验室和工程中心，申报未来网大科学中心。推进合工大智能制造技术研究院建设，争取设立国家电动汽车及分布式能源协同创新中心。国家、省市联手，推动语音产业快速发展。加强十大战略性新兴产业研究院建设，提升实体化、开放式运作水平。进一步完善“一中心、三基地”成果展示、创新服务等功能，加快二期工程建设。启动“中科智城”规划建设。

全面优化创新环境。完善深化科技体制改革、推进创新体系建设的政策措施，大力推进企业股权和分红激励试点，积极鼓励科技成果出资入股。加强知识产权保护，不断优化科技创新的法治环境。健全多元化科技创新投入机制，推进国家科技金融结合试点，积极争取高新区列入“新三板”扩容试点，引导发展一批产业专业投资基金。加强创新人才基地、载体建设，新建10家院士和博士后工作站。

（五）统筹城乡发展

全面建设美好乡村。加大投入，创新机制，高标准规划建设中心村150个，整治自然村1000个，改造农村危房12000户。积极谋划环巢湖流域土地整治重大工程，加快实施董铺和大房郢水库水源地、庐江汤池、新桥国际机场周边三大项目和10个国家级示范项目，完成土地整治20万亩。加强农村基础设施建设，完成水库除险加固51座、县乡道升级改造工程150公里、村村通提级延伸联网工程100公里、危桥加固改造35座。大力开展农村环境综合整治，创建一批星级乡镇。

加快县域经济发展。坚持工业立县主战略，积极承接产业转移，引导优势骨干企业快速扩张，吸引更多大项目落户，加快形成和壮大县域主导产业。制定支持政策，加快县域城镇化步伐，提升县城建设品位和管理水平，启动实施15个中心镇和三河、长临、下塘、中庙、汤池等5个镇级市规划建设，推进工业园区与城镇协同发展。按照等高对接、融合发展的要求，加大“十大工程”和“十大政策”的实施力度，加快巢湖华能电厂二期、庐江钒资源开发和综合利用、罗河铁矿等重大工业项目和半汤、汤池温泉旅游度假区建设，支持经开区、高新区等国家级开发区与巢湖、庐江开发园区合作共建一批投资大、就业多、效益好的标杆性大项目，全力推动巢湖、庐江迈出更快更大的发展步伐。

大力发展现代农业。新增特色高效农业10万亩，打造10个年产值超10亿元的特色产业集群。加快庐江郭河、肥东现代牧业、巢湖中粮综合体等现代农业园区建设，新增各类农业示范园区20个。加快培育新型农业经营主体，着力打造一批年销售收入10亿元以上的农业产业化龙头企业。大力推进农民专业合作社示范社建设，全面完善基层农技推广服务体系。

（六）深化改革开放

实施改革攻坚。加速国有企业战略重组步伐，优化国有资产布局，实现保值增值。强化政策支持，鼓励引导民间资本进入市政、教育、卫生、金融、环保、养老等领域，大力扶植一批非公有制骨干企业。深化地方金融体系改革，探索建立股权融资、融资担保、资本项目对接三大金融创新体系，壮大建投集团等政府基础设施投融资平台，完善兴泰控股等综合性金融产业平台，做优工投公司等新型工业服务平台，积极争取组建地方金融资产管理公司。加强政府债务管理，确保风险合理可控。巩固扩大“营改增”试点成效，推进税源专业化管理试点。深化招投标管理创新，积极构建区域性公共资源交易大市场。加快推进以“二标五权”为重点的农村产权制度改革，全面完成县级公立医院综合改革，稳步开展事业单位分类改革。

扩大对外开放。完善政策引领，依托“合肥之友”等招商平台，深化“百名县处级领导干部大招商”活动，瞄准世界500强、中国500强、行业100强、上市公司以及央企、知名民企，突出长三角、珠三角以及欧美、东南亚、港澳台等重点区域，开展高密度系列招商推介活动。更加注重定点招商、产业链招商、园区招商，提高招商活动的针对性和有效性。全年引资2510亿元、增长20%，其中，外商直接投资18.4亿美元、增长15%；引进工业大项目20个、现代服务业大项目30个。推进与美国富兰克林郡绿色合作伙伴计划，加强与哥伦布、戴瑞滨等国际友城的交往。加强区域合作发展，推进合肥经济圈建设，深化与阜阳市和六安市霍邱县、寿县结对合作，加快合肥阜阳现代产业园区、寿县蜀山现代产业园区建设。加大援疆、援藏工作力度。

（七）推进文化强市建设

繁荣文化事业。实施“文化基础设施提升工程”，坚持省市共建共享，打造一批标志性、基础性文化设施，积极服务省重大文化艺术项目建设。实施“文化惠民工程”，推进乡镇综合文化站、标准化档案室、农村电影“2131”、广播电视“村村通”和社区图书室、职工书屋、公共电子阅览室等建设。实施“文化品牌打造工程”，整合社会资源，推出一批在全国有影响的文化精品力作。

壮大文化产业。实施“文化产业跨越工程”，坚持重大项目带动，推进国家级文化和科技融合示范基地建设，加快文化产业园区发展，扶持市文广集团、报业集团、演艺集团等一批骨干企业做大做强。编制新兴文化产业发展规划，在文化创意、动漫游戏、数字出版、影视制作等方面加快打造一批特色产业集群。办好文博会、巢湖国际旅游节等文化会展活动。实施“文化发展环境优化工程”，创新工作推进机制，完善文化产业政策，加强人才引进和培养，造就一批文化领军人物和拔尖人才。

创建文明城市。以创建全国文明城市为目标，以提升市民素质为核心，全面深化群众性精神文明创建活动，扎实推进“三城同创”。大力建设社会主义核心价值体系，广泛开展道德模范、身边好人等先进典型的评选表彰和“三个倡导”宣传教育活动，积极创建全国未成年人思想道德建设先进城市。深入开展“文明劝导行”、“城管微博”等各类志愿服务活动，提升志愿服务常态化水平。加强信用体系建设，积极争取国家信用城市建设试点。创新网络出版监管模式，完善网络舆情监测体系。

（八）加强生态城市建设

建设环巢湖生态示范区。积极推进《巢湖流域管理条例》制定工作，加快编制环巢湖生态示范区建设规划。按照截污减负、增容复苏、修复共生“三大路径”，实施入湖口截污、生态修复、通江航道等“八大工程”。强化入湖河流综合治理，加快沿河沿湖岸边居民生态搬迁和湖滨带保护修复。大力推进环巢湖生态示范区一期项目建设，积极谋划二期项目，重点实施现有污水处理厂提标改造和国家规划的环湖污水处理厂建设，同步推进配套的400公里管网建设。加大水环境监管力度，实施巢湖“水专项”课题试验项目。加强矿产资源勘察保护和合理开发利用，加大矿山整治和生态修复力度，建设环巢湖地区矿山地质环境治理示范工程。

创建国家森林城市。大力实施“森林增长工程”，掀起新一轮植树造林热潮，完成植树造林33万亩、新增和提升绿化面积1200万平方米。大力推进江淮分水岭脊线、巢湖边岸等重点区域集中造林，加快机场高速、合安高速、各组团之间、十五里河等绿色生态廊道建设。扎实推进农村“五个一”绿化工程，深入开展城区绿化大会战，提高社会绿化水平。推进坝下河、丝绸、滨湖等7个新公园建设，扩建和提升环城、花冲、包公等6个公园。

加强节能减排和环境保护。大力推广绿色建筑等先进节能模式，积极开展能耗对标、合同能源管理活动，推进清洁生产，实施一批畜禽养殖减排等重点项目。加强扬尘治理，实时发布PM2.5监测数据，限行“黄标车”，严处“冒黑烟”机动车。加强垃圾无害化处理，建成生活垃圾焚烧发电厂，开工建设餐厨垃圾处理厂。加大固体废弃物处置力度，严格控制城市噪音污染。

（九）加强民生保障

提高居民收入。大力实施城乡居民收入倍增计划，深化收入分配制度改革，努力实现居民收入增长和经济发展同步、劳动报酬增长和劳动生产率提高同步。制定社会服务“四大政策”，出台实施基本公共服务体系“十二五”规划。扎实推进民生工程，强化后续管理，切实解决好人民群众最为期盼又带有普惠性、制度性的民生项目。加大扶贫开发力度，设立专项资金，着力构建产业扶贫、行业扶贫和社会扶贫新格局。

优先发展教育。完成学前教育“三年行动计划”，加大普惠幼儿园建设力度，新建、改扩建幼儿园45所。推进城乡义务教育均衡发展，新建、改扩建中小学10所。加快合肥七中新校区建设，启动合肥十中新校区建设。推进基础教育课程改革，提高素质教育水平。加快职教基地建设，大力推进幼儿师专、经贸旅游学校、工业学校二期工程建设。加强合肥学院、合肥职业技术学院建设，省市共建安徽艺术学院，支持中央、省在肥高校发展。

推动创业就业。实施就业优先战略，大力鼓励创业，完善扶持政策体系，重点做好高校毕业生、农村转移劳动力和退役军人就业工作，建成一批示范性创业孵化基地，帮助“零就业”家庭成员、“4050”人员、残疾人等困难人群实现再就业，全年新增城镇就业岗位12万个。强化职业技能培训，提升就业创业能力，增强就业稳定性。加强就业服务和管理，构建和谐劳动关系。

加强社会保障。完善城镇职工基本养老保险及城乡居民养老保险制度，切实保障失地农民、失业人员等低收入群体基本生活。加强社会救助体系建设，提高优抚对象、城乡低保、农村五保、医疗救助、生育保险等待遇水平，农村五保供养标准提高到每人每年不低于2200元。大力发展老龄服务事业和产业，建立完善居家养老为基础、社区养老为依托、机构养老为支撑的养老服务体系。实施安居工程，开工各类保障性住房和棚户区改造3.35万套，完成老旧小区环境综合整治120个，实施城中村和旧城改造40个。优化商品房供应结构，推进住宅产业化，促进房地产市场平稳健康发展。

推进全民健康。广泛开展爱国卫生运动，积极争创全国卫生城市。加强公共卫生服务体系建设，加大食品药品安全监管力度。完善城镇职工、居民基本医疗保险及大病救助制度，全面推进基本医疗保险门诊统筹。大力开展全民健身系列主题活动，打造“十分钟健身圈”，推动群众体育与竞技体育全面发展。加强人口和计划生育工作，巩固扩大全国流动人口计划生育基本公共服务均等化试点成果。

（十）促进社会和谐稳定

加强社会管理创新。创新手段和方式，改进流动人口和特殊人群管理服务，实行流动人口居住证统一管理，强化实有人口、实有房屋、社会组织、虚拟社会管理。加快城乡社区服务体系和信息化建设，推进社会管理精细化、高效化。大力培育新兴社会组织，提高非基本公共服务水平。全面推进社会稳定风险评估，深入开展矛盾纠纷排查化解，完善处理疑难复杂信访问题工作机制。支持工会、共青团、妇联等人民团体广泛参与社会管理及公共服务，扎实做好防震减灾、民族宗教、地方志、科普、气象、统计、保密、对台、侨务、社科等工作，推动慈善事业加快发展，积极创建“全国残疾人文化建设示范市”。

加强公共安全体系建设。加快实施“天网”、“地网”、“虚拟网”三网工程，继续大力整治“三无”小区和“五小行业”，强力推进打防管控一体化，积极构建数字化、智能化、立体式的社会治安防控体系。深入实施“六五”普法工作，提高法制宣传教育成效。加强应急救援队伍、装备和物资储备体系建设，增强应对突发事件能力。严格落实安全生产责任制，完善隐患排查治理体系，大力开展烟花爆竹企业退出、非煤矿山整顿关闭以及危险化学品、建筑施工、特种设备、道路交通、消防等专项整治，确保安全生产态势平稳。加强国防动员和民兵预备役建设，争创全国双拥模范城“八连冠”，全面推进人防民防“进社区”工作。

四、加强政府自身建设

面对新形势、新任务，我们要更加自觉地以科学发展观武装头脑，严格落实中央“八项规定”，坚持做到“讲大局、强责任、提能力、抓落实”，大力建设学习型、创新型、服务型政府。

进一步提升创新理政水平。坚持解放思想，善于用改革的办法、开放的思路和创新的举措破解发展难题。加强和改进调查研究，深入基层了解真实情况，准确把握客观发展规律，提高科学决策、民主决策能力。树立全球视野，强化战略思维，深刻认识战略机遇期内涵和条件的重大变化，因势利导，顺势而为。坚持开拓创新，以敢作为、勇担当的胆略，用好用活各项“先行先试权”，再创一批在全省乃至全国具有示范意义的“先行之作”。

进一步提升为民执政水平。自觉把人民放在最高位置，始终与群众心心相印、同甘共苦、团结奋斗。坚持问政于民、问需于民、问计于民，确保各项决策和工作更加符合群众意愿。深入推进效能革命，进一步深化行政审批制度改革，推行审批窗口首席代表制，完善重大投资项目全程代理制。整合政务服务资源，完善“12345政府服务直通车”运行机制，形成上下联动、层级清晰、覆盖城乡的政务服务体系，促进政务服务均等化、规范化、高效化。大力推行建设项目收费“一表制”，巩固完善建设工程竣工联合验收制度。严格绩效管理、行政问责，坚决治庸、治懒、治散。

进一步提升务实施政水平。弘扬真抓实干的工作作风，完善重大事项和重点工作跟踪落实机制，做到每一项工作任务都有责任领导和责任部门，每一个工作环节都有责任岗位和责任人员。牢固树立艰苦奋斗、勤俭节约的思想，严格控制“三公”消费，力戒奢靡之风，杜绝铺张浪费。精简会议和文件，开短会、讲短话、行短文。严格控制各类检查、评比，从简、务实举办各类展会和节庆活动。各级政府部门特别是领导干部要腾出更多的精力谋大事、谋发展，集中更多的时间抓具体、抓落实。

进一步提升依法行政水平。自觉接受人大法律监督和政协民主监督，认真办理人大代表议案、建议和政协提案，充分听取各民主党派、工商联、无党派人士及各人民团体的意见，广泛接受社会公众和新闻舆论监督。加强政府立法，继续做好规章、规范性文件清理工作。严格落实重大行政决策程序规定，全面推进行政处罚群众公议制度和行政首长出庭应诉工作。完善政务公开制度，健全新闻发布和发言人制度，拓宽电视、广播、报纸、网络“四位一体”的政民互动渠道。

进一步提升廉洁从政水平。全面推进惩治和预防腐败体系建设，严格落实党风廉政建设责任制。加强领导干部经济责任审计，完善反腐倡廉举报受理机制，严肃查处工程建设、土地管理、政府采购、产权交易和行政审批等领域违法违纪案件。深化重点领域专项治理，坚决纠正损害群众利益的不正之风。完善纪检监察“全派驻”和重大项目纪检监察派驻制度。健全教育培训、选调交流、激励约束等机制，着力打造一支想干事、会干事、干成事的公务员队伍。

各位代表！

实现科学发展新跨越任重道远，建设幸福美好新合肥催人奋进。让我们紧密团结在以习近平同志为总书记的党中央周围，在省委、省政府和市委的坚强领导下，紧紧依靠全市人民，继往开来，团结奋进，为实现“新跨越、进十强”，打造“大湖名城、创新高地”，加快建设现代化新兴中心城市和区域性特大城市而努力奋斗！
